# Fair ranking of researchers and research teams

**DOI:** 10.1371/journal.pone.0195509

**Published:** 2018-04-05

**Authors:** Václav Vavryčuk

**Affiliations:** Institute of Geophysics, The Czech Academy of Sciences, Prague, Czech Republic; CPERI, GREECE

## Abstract

The main drawback of ranking of researchers by the number of papers, citations or by the Hirsch index is ignoring the problem of distributing authorship among authors in multi-author publications. So far, the single-author or multi-author publications contribute to the publication record of a researcher equally. This full counting scheme is apparently unfair and causes unjust disproportions, in particular, if ranked researchers have distinctly different collaboration profiles. These disproportions are removed by less common fractional or authorship-weighted counting schemes, which can distribute the authorship credit more properly and suppress a tendency to unjustified inflation of co-authors. The urgent need of widely adopting a fair ranking scheme in practise is exemplified by analysing citation profiles of several highly-cited astronomers and astrophysicists. While the full counting scheme often leads to completely incorrect and misleading ranking, the fractional or authorship-weighted schemes are more accurate and applicable to ranking of researchers as well as research teams. In addition, they suppress differences in ranking among scientific disciplines. These more appropriate schemes should urgently be adopted by scientific publication databases as the Web of Science (Thomson Reuters) or the Scopus (Elsevier).

## Introduction

The simplest way how to measure the quality of scientists is to evaluate the following three integer numbers: the number of published papers, the number of citations, and the *h*-index introduced by Hirsch [1] and defined as the maximum number of papers of a scientist which are cited at least *h* times. Although ‘*a single number can never give more than a rough approximation to an individual's multifaceted profile*, *and many other factors should be considered in combination in evaluating an individual*’ [1], it is believed that this metric provides a useful measure of the productivity of a scientist and the impact of his research. In particular, the *h*-index has become popular and widely accepted, because it reflects both the quality and quantity of the scientific output. It smartly suppresses the disproportionate weight of a few highly cited papers as well as it ignores less significant papers with no or few citations. The *h*-index is usually determined as an integer but it might be modified to be real valued [2]. Also other generalizations or modifications of the *h*-index have been proposed by some authors [3–9] including a specification which type of papers (e,g., peer-review papers, proceedings, book chapters) should be considered for ranking [10].

Obviously, research evaluation and the definition of ranking criteria have an impact on the research itself in a long-term prospect. Researchers try to increase their ranking by complying with the presently accepted criteria. As a result, the number of citations is being increased by self-citations and coercive citations [11], and the number of published papers and the *h*-index are rising over time by the inflated number of multi-author publications and the number of authors [12–13]. As discussed by Papatheodorou et al. [14], the inflation of authors is not just due to an increasing research complexity but it is also shaped by the interplay of ‘publish or perish’ pressures, collaborative needs and the visibility of research. Kwok [15] also discusses an unethical behaviour of some scientists who required the co-authorship to get better ranking.

In this paper, I focus on the problem how to deal properly with multi-author papers in ranking researchers and research teams. I review several alternative approaches to the standard ranking scheme and discuss their pros and cons. Analysing synthetic examples as well as citation profiles of several highly-cited astronomers and astrophysicists I expose the failure of the standard ranking criteria and point to urgent need for adopting a more accurate and fairer ranking scheme in the evaluation practise. I show that the standard ranking can lead to completely incorrect and misleading evaluations. The behaviour and trends of team ranking in dependence of the quality and the number of individual team members is also discussed.

## Authorship counting

### Full counting

The main drawback of ranking of scientists by the total number of papers, citations or by the standard *h*-index is ignoring the problem of co-authorship in multi-author publications. So far, single-author or multi-author publications contribute to the publication record of a scientist equally [1]. This full counting method is very simple and easy to apply but it is apparently unfair and causes unjust disproportions in the evaluation [12–14]. Obviously, individual contributions of ten co-authors to a paper are very different than if the paper is written by a single author. Moreover, papers with ten or more co-authors are not exceptional; some papers have even more than 500 co-authors [16]. For example, a physics paper with more than 5000 authors was published in 2015 [17]. Since various research fields are characterized by a different extent of collaboration, the full counting produces also significant differences among scientific disciplines [18].

### Fractional counting

The fractional counting considers the number of papers fractionally according to the number of authors [18–20]. For example, Batista et al. [18] substitute the *h*-index by the index *h*_*I*_ = *h*^2^/*N*_*a*_, where *N*_*a*_ is the total number of authors in the considered *h* papers. Another possibility, proposed by Schreiber [21–22] and Egghe [4], is to distribute uniformly the authorship credit among authors for individual papers. For example, three authors of a paper receive equally one third of the authorship credit. Both approaches [18,21–22] yield similar ranking which removes evident disproportions in author’s contributions in single- or multi-author papers and thus represents a significant improvement of the original *h*-index. Moreover, this counting removes quite effectively differences among various scientific disciplines [18].

Nevertheless, some authors argue that this scheme: (1) discourages a collaboration, which is essential for progress in science, and (2) divides the credit equally, which is not necessarily accurate and can lead to neglecting a crucial role of some co-authors [23]. However, as mentioned by Waltman & Van Eck [24] these arguments are not fully justified because:

- The primary goal of ranking is to evaluate the scientific credit of researchers but not their collaboration abilities. If needed, the collaboration of a researcher can be quantified independently by the mean number of co-authors per paper or by the *c*-index, defined in analogy to the *h*-index but instead of counting citations we count the number of co-authors.
- A fruitful collaboration results in publishing a high number of high-quality papers (i.e., more papers with more citations), so the co-authors benefit from a productive collaboration even under the fractional scheme.
- The distribution of the equal credit among all authors cannot be taken as an argument for preferring the full counting over the fractional counting, because the equal-credit distribution is common to both schemes. Moreover, the equal-credit distribution can easily be removed by modifying the simple fractional scheme to more sophisticated authorship-weighted schemes which can better reflect contributions of the individual authors.

### Authorship-weighted counting

This type of counting attempts to distribute credit between authors more properly than the simple fractional counting. The authorship credit of each paper is usually assumed to have a value of 1 and it is split according to various rules attempting to quantify contributions of individual authors. I review several possible schemes how to distribute the authorship among the co-authors [25–27]:

- The ‘*equal-contribution*’ (EC) scheme, when the authorship credit is distributed among all authors equally. This is the standard fractional scheme [4,21–22], being appropriate to papers when the authors use the alphabetical sequence to emphasize a similar contribution in the collaborating group.
- The ‘*sequence-determines-credit*’ (SDC) scheme, when the sequence of authors reflects declining importance of the co-author’s contribution. This scheme is appropriate if the authors do not use the alphabetical sequence and the number of authors is not too large. The first author is the main contributing author and receives the highest credit. The credit of the other authors gradually decreases with the position in the list. The distribution of credits among the authors can be calculated using harmonic counting [28–31], geometric counting [25], arithmetic counting [30] or other counting methods [8].
- The ‘*first-author-emphasis*’ (FA) scheme, when the first author as the main contributor has higher credit than the other co-authors. In an extreme case, the first author can receive the full credit and the other authors no credit [32–33]. A more appropriate approach is, however, to allocate only some limited bonus to the first author. The other authors receive either equal credit as in the EC scheme or gradually decreasing credit as in the SDC scheme [34].
- The ‘*first-last-author-emphasis*’ (FLA) scheme, when the first author as the main contributor and the last author as the project leader have higher credits than the other co-authors [26,35–36]. This approach is, however, somewhat confusing because it mixes scientific credit with leadership abilities. Similarly as with quantifying the success in collaboration, the success in a research leadership and in supervision of young researchers should be evaluated separately by another factor.
- The ‘*corresponding-author-emphasis*’ (CA) scheme, when an extra credit is allocated to the corresponding author [24,33–34,37–40]. This scheme is suitable particularly when the authors are listed in the alphabetical sequence.
- The ‘*contribution-indicated*’ (CI) scheme, when individual contributions are explicitly acknowledged by authors themselves according to the policy of some journals.

## Combined weighted counting scheme

The variety of counting schemes for evaluating the authorship indicates a complexity of the problem. It is evident that, except for the CI scheme, no other scheme is fully accurate and general [26,41]. Since the CI scheme is not applicable to all papers at present, it is desirable: (1) to find and accept some compromise for measure of the authorship, and (2) to know how sensitive is ranking of researchers to the applied counting scheme. For this purpose, I propose a simple authorship-weighted scheme which combines basic features of the most important weighted schemes listed in the previous section and compare the *h*-index calculated by this scheme with the full and fractional counting schemes.

The combined weighted scheme is defined as follows:

- The sum of authorships of individual authors equals 1 for each paper. This is a basic condition which ensures that all papers have an equal weight irrespective of the number of co-authors. This condition is crucial for fair ranking of researchers and it is violated in the standard full ranking scheme.
- If possible, the authorship is defined by the authors themselves (CI scheme).
- In the other cases, the authorship is allocated as follows:
  ○ If the authors are listed in alphabetical order and the corresponding author is indicated, then the corresponding author receives a bonus and the rest is equally divided into all co-authors. If the bonus is zero, we get the simple fractional scheme. For bonus *b* = 20%, the authorship is 6/10 and 4/10 for two authors, and 7/15, 4/15 and 4/15 for three authors, for other examples see Tables 1 and 2. If the corresponding author is not indicated, the full authorship is equally divided into all co-authors.
  ○ If the authors are not listed in an alphabetical order, the first author and the corresponding author receive the same bonus *b* and the rest is equally divided into all co-authors. If the first author is also the corresponding author, the total bonus is 2*b*. Hence, for bonus *b* = 20%, the authorship is 7/10 and 3/10 for two authors, in the case that the first author is also the corresponding author, but 5/10 and 5/10 if the corresponding author is the second author. For other examples, see Tables 1 and 2.
  ○ In the case of several corresponding authors, the bonus *b* is split equally among them. The same applies to several equal-first authors, which might also occasionally occur [42].

**Table 1:** Authorship weights for the combined counting scheme with bonus *b* = 20%.

	Alphabetical order		Non-alphabetical order F = C		Non-alphabetical order F≠C		
authors	C	O	F = C	O	F	C	O
2	0.600	0.400	0.700	0.300	0.500	0.500	---
3	0.467	0.267	0.600	0.200	0.400	0.400	0.200
4	0.400	0.200	0.550	0.150	0.350	0.350	0.150
5	0.360	0.160	0.520	0.120	0.320	0.320	0.120
6	0.333	0.133	0.500	0.100	0.300	0.300	0.100
10	0.280	0.080	0.460	0.060	0.260	0.260	0.060
100	0.208	0.008	0.406	0.006	0.206	0.206	0.006

C–the corresponding author, F–the first author, F = C–the first author is also the corresponding author, O–the other author(s).

**Table 2:** Authorship weights for the combined counting scheme with bonus *b* = 30%.

	Alphabetical order		Non-alphabetical order F = C		Non-alphabetical order F≠C		
authors	C	O	F = C	O	F	C	O
2	0.650	0.350	0.800	0.200	0.500	0.500	---
3	0.533	0.233	0.733	0.133	0.433	0.433	0.133
4	0.475	0.175	0.700	0.100	0.400	0.400	0.100
5	0.440	0.140	0.680	0.080	0.380	0.380	0.080
6	0.417	0.117	0.667	0.067	0.367	0.367	0.067
10	0.370	0.070	0.640	0.040	0.340	0.340	0.040
100	0.307	0.007	0.604	0.004	0.304	0.304	0.004

C–the corresponding author, F–the first author, F = C–the first author is also the corresponding author, O–the other author(s).

This counting scheme is simple and similar to the fractional counting of Schreiber [21–22,43] except for a bonus for the first and corresponding authors. Allocating a bonus to the first author in a non-alphabetical authorship reflects the principal role of this author. Allocating a bonus to the corresponding author in alphabetical and non-alphabetical authorships is desirable for several reasons. First, one of the authors has always a major contribution in preparing the paper, even in the case of publications with the alphabetical authorship. Second, it might happen that the alphabetical authorship is not intentional, in particular, when the number of authors is low [11]. In this case, the first author can get a bonus as the corresponding author. Third, the scheme is also able to distribute credits between young authors and supervisors and to emphasize the role of group leaders, who can receive an extra credit as the corresponding author(s). The value of the bonus for the first and corresponding authors should be between 10% and 40%. A low value of the bonus suppresses a role of principal contributors, while a high value of the bonus causes almost negligible authorship of the other authors. The authorship distributions for *b* of 20% and 30% are summarized in Tables 1 and 2.

## Mathematical definition of the weighted scheme

The following series of numbers are needed for quantifying the publication career of a scientist:

- ‘rank-citation profile’ *c*_*r*_, *r* = 1,…,*N*, which is the number of citations to his/her paper *r* ranked in the decreasing order,
- ‘author-number profile’ *n*_*r*_, *r* = 1,…,*N*, which is the number of authors of his/her paper *r*,
- ‘authorship profile’ *a*_*r*_, *r* = 1,…, *N*, which quantifies the authorship percentage of his/her paper *r*,
- ‘cumulative authorship profile’ $A_{r}=\sum_{i=1}^{r}a_{i}$, *r* = 1,…,*N*, which cumulatively sums the authorship of ranked papers,

where *N* is the total number of papers published by a given scientist.

The authorship *a* of a paper is calculated for the alphabetical order of authors as

$$a_{}=\frac{1}{n}\left(1-b\right)+bA_{}^{C},$$ (1)

and for the non-alphabetical order of authors as

$$a_{}=\frac{1}{n}\left(1-2b\right)+b\;\left(A_{}^{F}+A_{}^{C}\right),$$ (2)

where *n* is the number of authors of the paper, *b* is the bonus, *A*^*F*^ is 1 for the first author and 0 for the other authors, and *A*^*C*^ is 1 for the corresponding author and 0 for the other authors. The bonus *b* can range from 0 (the simple fractional scheme) to 0.5 (full credit is distributed between the first and corresponding authors). Eqs (1) and (2) ensure that the sum of authorships of all authors of any individual paper equals 1.

Considering the authorship-weighted scheme, the number of published papers *N* and the number of citations *C* is replaced by the weighted number of papers *N*_*W*_ and the weighted number of citations *C*_*W*_:

$$N_{W}=\sum_{r=1}^{N}a_{r},$$ (3)

$$C_{W}=\sum_{r=1}^{N}a_{r}c_{r}.$$ (4)

Furthermore, the original *h*-index defined as

$$h=\underset{r}{\max}\left(r\leq c_{r}\right)$$ (5)

is replaced by the ‘authorship-weighted’ (or simply ‘weighted’) *h*_*W*_-index defined as

$$h_{W}=\sum_{i=1}^{r_{W}}a_{i},$$ (6)

where *r*_*W*_ is the number of papers contributing to the *h*_*W*_-index

$$r_{W}=\underset{r}{\max}\left(\sum_{i=1}^{r}a_{i}\leq c_{r}\right).$$ (7)

**Fig 1:**
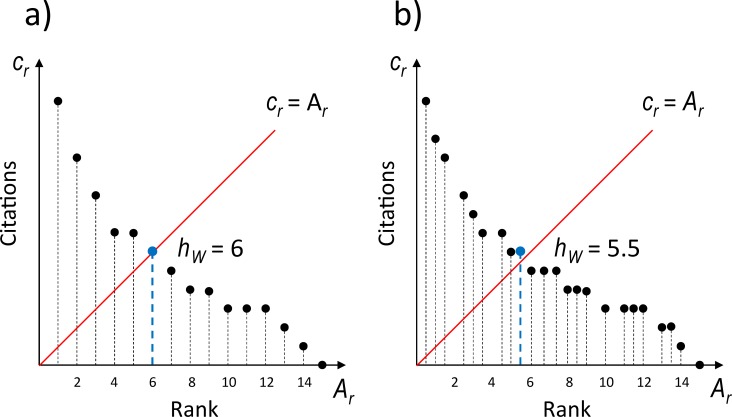
**Definition of the *h***_***W***_**-index for (a) single-author publications, and (b) a mix of single- and multi-author publications.** Quantity *c*_*r*_ is the rank-citation profile of a scientist (or simply ‘citations’), *A*_*r*_ is the corresponding cumulative authorship profile of published papers (or simply ‘rank’). The blue dot shows the threshold value controlling the *h*_*W*_-index. The *h*_*W*_-index in (a) is identical with the standard *h*-index.

The weighted publications, citations and the *h*_*W*_-index are no longer integers but positive real numbers. The meaning of the *h*_*W*_-index is graphically illustrated in Fig 1. For rank-citation profiles with single-author publications only, the *h*-index and the *h*_*W*_-index yield identical values. For highly collaborative authors, both indices can be remarkably different. The collaboration of authors might be quantified using the *collaboration index c*:

$$c=\underset{r}{\max}\left(r\leq {\bar{n}}_{r}\right),$$ (8)

where ${\bar{n}}_{r}$ is the author-number profile *n*_*r*_ sorted in the descending order. The definition of the *c*-index is analogous to that of the *h*-index, so the *c*-index of 5 means that a researcher published 5 papers with at least 5 authors. The *c*-index in (8) is defined using the whole author-number profile, but it can also be restricted to the papers which contribute to the original or weighted *h*-index [18]. Research teams or research institutions can be ranked in a similar way as individual researchers. The weighted numbers of publications and citations of a research team formed by *M* scientists can be obtained by summing the weighted numbers of publications and citations and the weighted $h_{W}^{team}$-index is calculated analogously as for individual members of the team

$$h_{W}^{team}=\sum_{i=1}^{r_{W}}a_{i}^{team},$$ (9)

where $r_{W}^{team}$ is the number of papers contributing to the $h_{W}^{team}$-index

$$r_{W}^{team}=\underset{r}{\max}\left(\sum_{i=1}^{r}a_{i}^{team}\leq c_{r}^{team}\right),$$ (10)

where $c_{r}^{team}$ is the rank-citation profile of the team, which is formed by gathering rank-citation profiles of its individual members and ordered in the decreasing sequence, and $a_{r}^{team}$ is the corresponding authorship profile.

## Synthetic example

Ranking of researchers and research teams is illustrated on the following example. We assume three research groups A, B and C, each with 10 researchers. For simplicity, the researchers have an identical rank-citation profile, *c*_*r*_ = (70, 50, 35, 25, 20, 17, 15, 12, 11, 10, 9, 7, 6, 4, 3, 2, 2, 1, 1, 0). Hence, each researcher is an author/co-author of 20 publications with the total number of 300 citations. As indicated by *c*_*r*_, the most cited paper has 70 citations and the least cited paper has no citations. The A-researchers are single authors, the B-researchers publish papers of 5 co-authors and the C-researchers publish papers of 10 co-authors (see Table 3). Hence the productivity of the A researchers is 5 times higher than that of the B researchers and 10 times higher than that of the C researchers. For papers with 5 co-authors, 3 co-authors are external (i.e., they are not members of the team); for papers with 10 co-authors, 8 co-authors are external. Author’s names in all multi-author papers are in the alphabetical order. The corresponding authors are the external researchers.

**Table 3:** Standard, fractional and authorship-weighted ranking of researchers.

Researcher	Authors per paper	Standard ranking			Fractional ranking			Authorship-weighted ranking		
		*N*	*C*	*h*	*N*_*m*_	*C*_*m*_	*h*_*m*_	*N*_*W*_	*C*_*W*_	*h*_*W*_
A	1	20	300	10	20	300	10	20	300	10
B	5	20	300	10	4	60	3	3.2	48	2.4
C	10	20	300	10	2	30	1.7	1.6	24	1.4

*N*, *C* and *h* are the number of papers, the number of citations and the standard Hirsch index of a researcher, *N*_*m*_, *C*_*m*_ and *h*_*m*_ are the corresponding quantities obtained using simple fractional (multi-author) counting, and *N*_*W*_, *C*_*W*_ and *h*_*W*_ are the corresponding authorship-weighted quantities (a bonus of 0.2 is considered for external corresponding authors).

**Fig 2:**
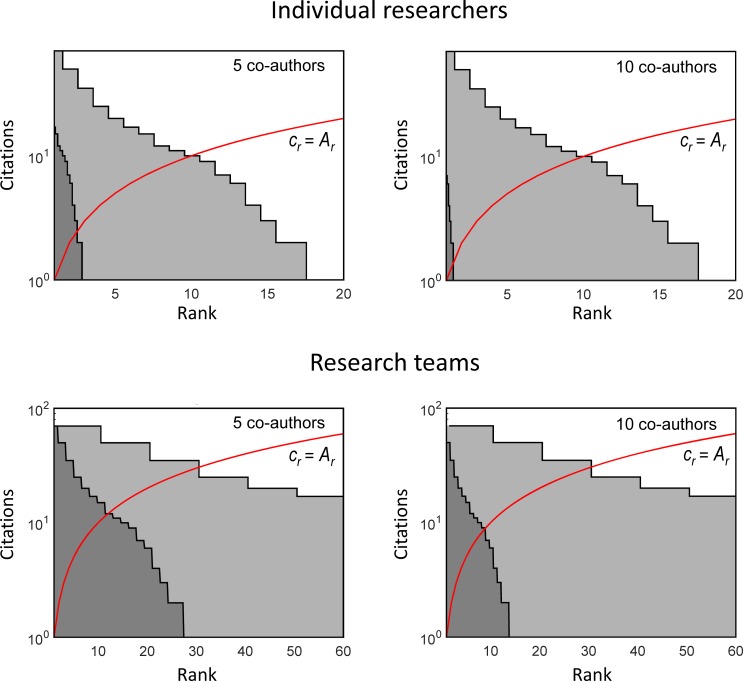
**The rank-citation profiles for individual researchers (upper panels) and research teams (lower panels).** Left: the B-researchers, right: the C–researchers. Light grey colour–full counting, dark grey colour–authorship-weighted counting. The red line marks the threshold defining the *h*-index. The plots are analogous to those in Fig 1 except for the citations axis, which is logarithmic. Consequently, the threshold line becomes curved.

Fig 2 (upper panels) and Table 3 illustrate the differences between the full, fractional and authorship-weighted counting in ranking of researchers (*h*-index, *h*_*m*_-index and *h*_*w*_-index, respectively). The full counting yields the same *h*-index for researchers of all three teams irrespective of the actual work load of researchers for producing the papers. By contrast, the fractional and authorship-weighted counting is sensitive to the number of co-authors of published papers. Obviously, the more co-authors of papers, the lower contribution of these papers to ranking is received. This is reflected in publications *N*_*W*_ (or *N*_*m*_), citations *C*_*W*_ (or *C*_*m*_) and the *h*_*W*_-index (*h*_*m*_-index). Fig 2 (lower panels) demonstrates the confusing results produced by applying the full counting criteria to evaluating research teams. Even though, team C has actually a twice lower weighted number of publications and citations than team B, the values of the full counting are identical for both teams. By contrast, the authorship-weighted quantities distinguish between the productivity of all three teams more properly.

**Fig 3:**
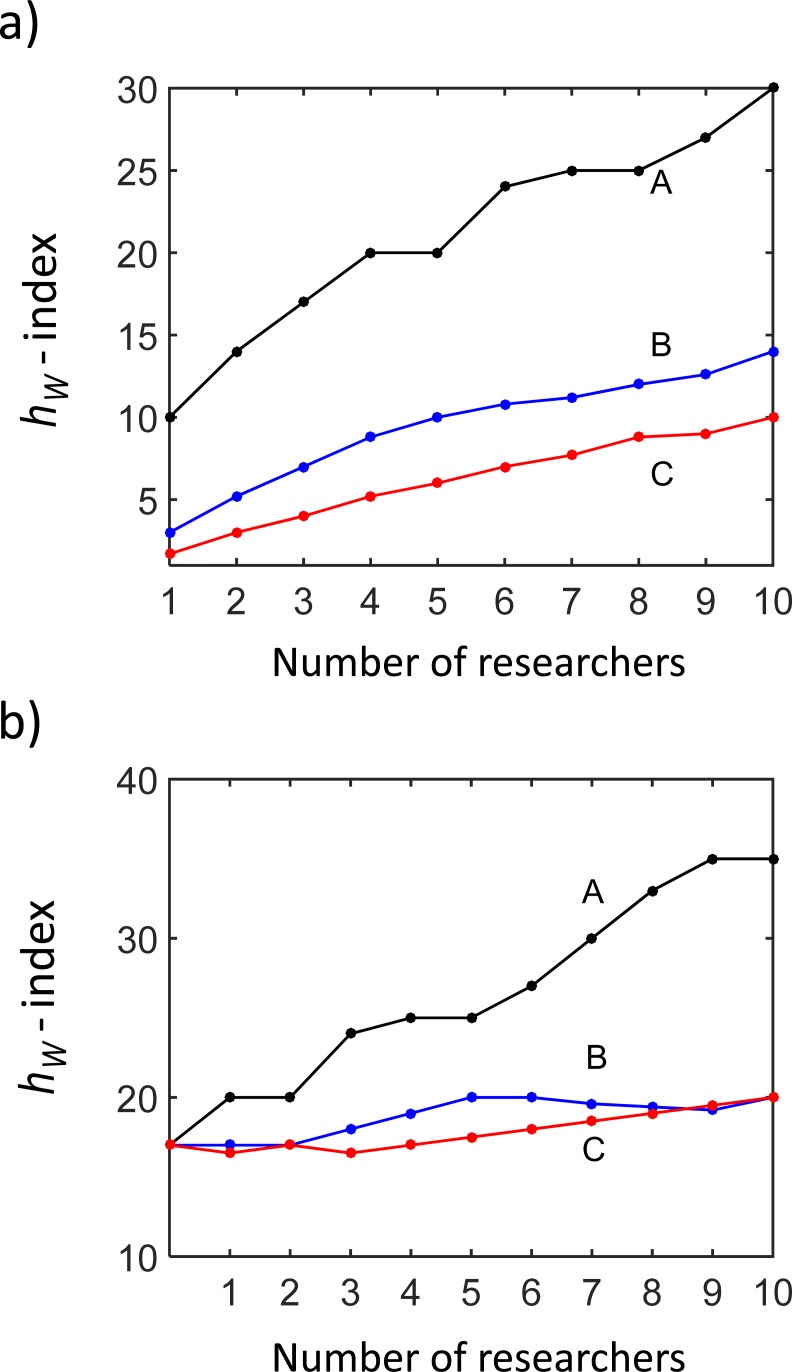
The team *h*_*W*_-index as a function of the number of researchers in the team. (a) The teams are formed by researchers with identical citation profiles: A (black line), B (blue line) or C (red line), respectively. (b) The teams are initially formed by three core A-researchers. The teams are further gradually extended either by the A-researchers (black line), the B-researchers (blue line) or the C-researchers (red line).

Calculating the *h*_*W*_-index for teams with a varying number of researchers, we can also address a problem how to build a team with the highest index. Fig 3 shows the team *h*_*W*_-index as a function of the number of the A-, B- and C-researchers in the team for two scenarios. First, we assume teams formed and gradually extended by including either A-, B- or C-researchers. So the teams are homogeneous consisting of researchers with the same authorship profile. Second, the teams have initially three A-researchers (single-author researchers) who form the core of the teams, and the teams are then extended by including either A-, B- or C-researchers. Hence, the initial *h*_*W*_-index of the core of the teams is equally 17.

In the case of the homogeneous teams, the *h*_*W*_-index linearly increases with the number of researchers (Fig 3A). The rate of the increase is, however, different: the single authors improve the team *h*_*W*_-index with the steepest rate. If including researchers, who published papers with a higher number of co-authors, the team *h*_*W*_-index increases with a lower rate. In the case of teams formed initially by three core A-researchers, the contributions of including the A-, B- or C-researchers to the team ranking are even more distinct. The increase of the team *h*_*W*_-index is much lower when extending the teams by the B- or C-researchers. It might even happen that including a new B- or C-researcher does not change the team index or the team index can slightly decrease (Fig 3B, red and blue curves).

## Ranking of selected astronomers and astrophysicists

The differences between the standard full counting and the authorship-weighted counting schemes are exemplified on selected highly-cited researchers working in astronomy and astrophysics. This scientific discipline is particularly suitable for this purpose because it offers a variety of research profiles from rather single authors to highly collaborative authors publishing as members of large research teams. We selected the following 9 reputed researchers: M. Colless, B.T. Draine, A.V. Filippenko, S.W. Hawking, Z. Ivezic, J.A. Peacock, P.J.E. Peebles, K.S. Thorne, and D.G. York, who have the *h*-index in the range from 66 to 109 and the collaboration index (*c*-index) from 3 to 95, according to the Web of Science (WOS) in December 2016, see Table 4. The researchers with a low *c*-index are mainly theorists (Hawking, Peebles), while those with a high *c*-index are partially or dominantly involved in large-scale experiments (Ivezic, Thorne, York). The selection of the researchers is subjective with no intention to produce statistically relevant results applicable to all researchers in astronomy and astrophysics. The data sample is designed just to exemplify how large differences can appear between various ranking schemes for researchers with a high *h*-index but with a diverse *c*-index.

**Table 4:** Standard, fractional and authorship-weighted ranking of selected astronomers and astrophysicists.

Researcher	Standard ranking					Fractional and authorship-weighted ranking		$h-h_{W}^{20}$	$h_{m}-h_{W}^{20}$
	*N*	*N*_1_	*C*	*h*	*c*	*h*_*m*_	$h_{W}^{20}$		
Peebles, P.J.E.	236	87	23x10^3^	70	4	57.3	54.4	15.6	2.9
Hawking, S.W.	137	94	31x10^3^	72	3	51.4	53.1	18.9	-1.7
Filippenko, A.V.	741	40	69x10^3^	107	38	44.7	40.3	66.7	4.4
Draine, B.T.	268	81	27x10^3^	73	24	42.4	43.0	30	-0.6
York, D.G.	489	23	58x10^3^	109	43	36.9	32.0	77	4.9
Thorne, K.S.	216	44	15x10^3^	66	95	32.6	29.9	36.1	2.7
Peacock, J.A.	246	26	47x10^3^	83	36	27.6	25.5	57.5	2.1
Ivezic, Z.	325	23	55x10^3^	107	40	18.0	16.7	90.3	1.3
Colless, M.	236	21	18x10^3^	67	30	15.1	14.4	52.6	0.7

*N* is the total number of papers, *N*_1_ is the number of papers with the first authorship, *C* is the number of citations, *h* is the standard Hirsch index, *c* is the collaboration index (for the definition, see Eq 7), *h*_*m*_ is the modified *h*-index obtained using the simple fractional counting, and $h_{W}^{20}$ is the authorship-weighted index with bonus *b* of 20%. The data are taken from the Web of Science (December, 2016).

**Fig 4:**
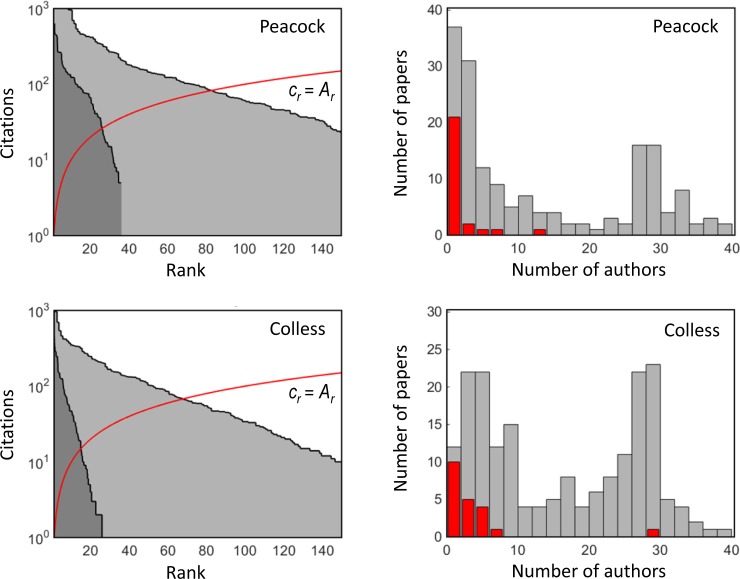
**The rank-citation profiles (left) and the collaboration profiles (right) for Peacock and Colless.** Left: light grey colour–full counting, dark grey colour–authorship-weighted counting. The red line marks the threshold defining the *h*-index. The axis showing the number of authors is clipped at the maximum value of 40 authors. The collaboration profiles distinguish whether the researcher is the first author (in red) or not (in grey). The data are taken from the Web of Science (December, 2016).

**Fig 5:**
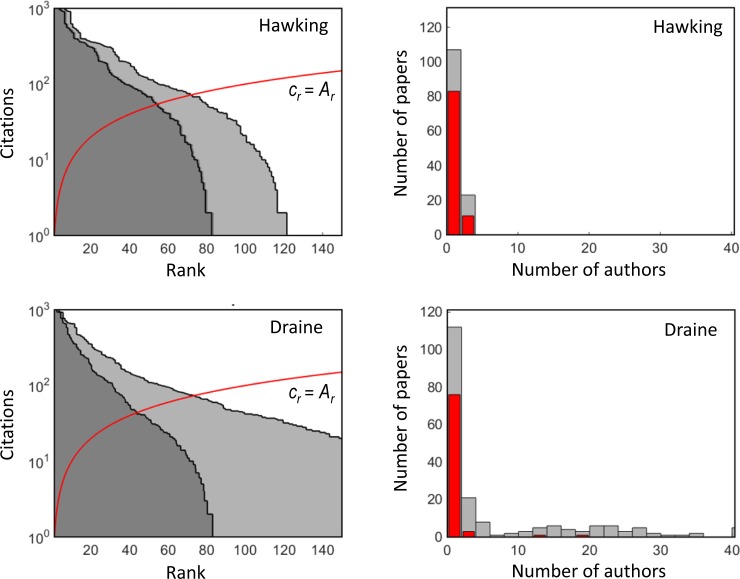
**The rank-citation profiles (left) and the collaboration profiles (right) for Hawking and Draine.** For details, see the caption of Fig 4.

Figs 4 and 5 show the rank-citation profile and the histogram of the number of papers as a function of the number of authors (collaboration profile) of four selected researchers. The profiles show that the differences between the individual researchers are substantial. For some researchers, the frequency of the number of co-authors has distinct peaks caused by a high number of publications reporting results of a specific large-scale experiment (e.g., papers with 28–30 co-authors in histograms of M. Colless and J.A. Peacock are related to the 2dF Galaxy Redshift Survey, see http://www.2dfgrs.net/). The collaboration profiles are clipped at the maximum number of 40 co-authors, but some researchers published papers with a remarkably higher number of co-authors.

Since the number of co-authors is fully ignored in the standard *h*-index, its value is overestimated for highly collaborative researchers. This is recognized when the more appropriate authorship-weighted ranking scheme is applied. Figs 4 and 5 (left-hand panels) show a comparison of both ranking schemes. The reduction in the *h*-index is enormous for some researchers. The differences between the full, fractional and authorship-weighted counting are summarized in Table 4. The authorship-weighted ranking is calculated for bonus *b* of 20% of the authorship, which is equally considered for the first and corresponding authors. Table 4 indicates:

- If the full counting is substituted by the authorship-weighted counting, the reduction of the *h*-index ranges from 15.6 to 90.3. This corresponds to a relative reduction of 23% to 84% of the original *h*-index. The highest discrepancy is for Z. Ivezic, when the ranking drops from 107 to 16.7. Similar values are obtained when the full counting is substituted by the fractional counting.
- The full counting scheme completely fails for 5 of 9 researchers, the difference between the standard and weighted *h*-index being higher than 60%.
- The differences between the simple fractional scheme and the authorship-weighted scheme are rather minor being 4.4 at most. Calculations not shown here indicate that the authorship-weighted ranking usually decreases with increasing bonus except for S.W. Hawking and B.T. Draine who published a high number of papers as the first authors.

**Fig 6:**
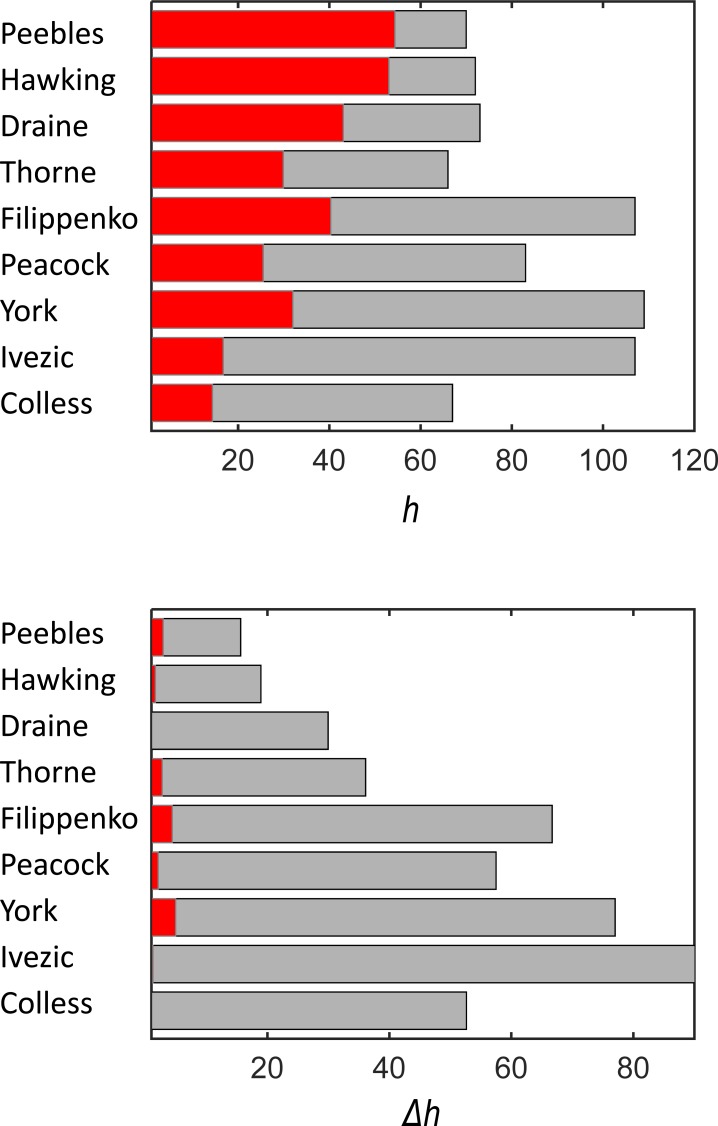
A comparison of the standard *h*-index and the fractional and authorship-weighted indices for selected highly-cited astronomers and astrophysicists. Upper panel: the standard *h*-index (grey colour) and the authorship-weighted *h*_*W*_-index (red colour). Lower panel: differences between the standard *h*-index and the authorship-weighted *h*_*W*_-index (grey colour) and between the fractional *h*_*m*_-index and the authorship-weighted *h*_*W*_ -index (red colour). Absolute values of the differences are shown. The lower panel indicates a good consistency between *h*_*m*_ and *h*_*W*_.

The discrepancy between the standard and authorship-weighted ranking is illustrated in Fig 6 (upper panel). The differences between the *h*-index and *h*_*W*_-index are so high for some researchers that the *h*-index cannot be considered even as a rough indication of the quality of a researcher. The standard ranking is simply wrong. Fig 6 (lower panel) shows a comparison of the differences between the standard and weighted ranking (in grey colour) and the differences between the weighted and fractional ranking (in red colour). Small differences between the fractional and weighted schemes confirm that: (1) applying even the simple fractional scheme leads to a significant improvement of the *h*-index, and (2) developing more complicated authorship-weighted schemes [8,23,30,39–40,44–45] than those analysed here is not very reasonable because the corrections will be minor.

**Fig 7:**
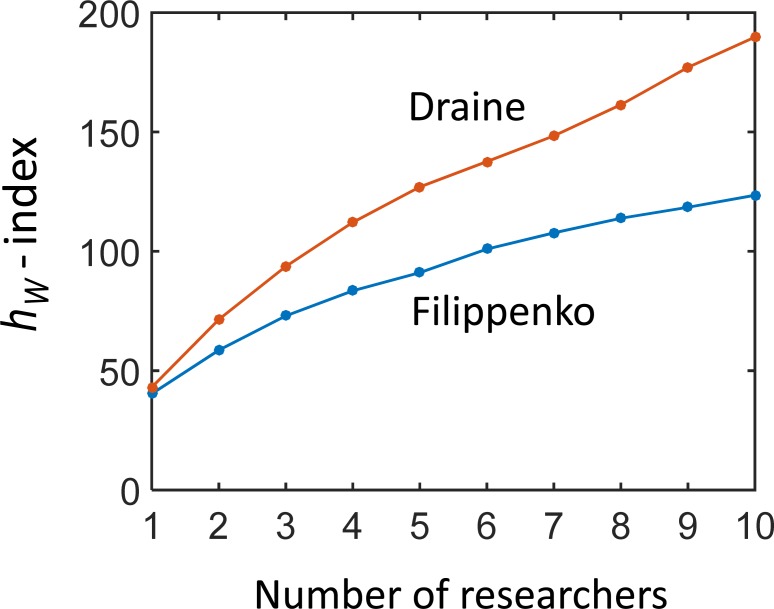
The team *h*_*W*_-index as a function of the number of researchers in two teams. The teams are formed by researchers with identical citation profiles of Draine and Filippenko.

Finally, we calculate the *h*_*W*_-index for teams with a varying number of researchers who have citation and authorship profiles identical with those of the selected astronomers and astrophysicists. Fig 7 shows an increase of the team *h*_*W*_-index with the number of team members characterized by two different citation profiles. Similarly as in the synthetic example (Fig 3), the less collaborative researchers contribute more to the team index than the more collaborative researchers. Even though the *h*_*W*_-index of the less and more collaborative researchers is similar, the increase of the team index with the number of researchers can be remarkably different.

## Discussion and conclusions

The current policy of evaluating the scientific output adopted by the Web of Science (Thomson Reuters) or Scopus (Elsevier) databases is unsatisfactory, because it ignores the problem of the authorship in multi-author publications. Although, many authors pointed to this controversy in ranking of scientists and proposed alternative schemes [4,16,21–22,26–27,36,43–44,46–47], the common practise has not been changed. This is unfortunate because a fair or unfair ranking has a feedback effect on science. Fair ranking of researchers can positively influence their publication habits. A fair distribution of authorship among co-authors can automatically suppress a tendency to unjustified inflation of co-authors because the authors will be more reluctant to share the authorship with colleagues not truly involved in the research or in preparing publications. The fair distribution of authorship will also remove existing evident disproportions between ranking of more and less collaborative researchers.

The synthetic examples and the analysis of real data show that substituting the full counting by the fractional or authorship-weighted counting systematically reduces the *h*-index of researchers and research teams. This reduction varies from 20% to 80% for the selected highly-cited astronomers and astrophysicists characterized by the collaboration index from 3 to 95. The *h*-index is reduced from 70 to 55 (Peebles), but also from 107 to 17 (Ivezic). These enormous disproportions point to a complete failure of ranking based on the full counting if applied to researchers with a high collaboration index. The disproportions are removed by applying a more appropriate counting scheme such as the fractional or authorship-weighted scheme. Applying the fractional scheme is elementary and the improvement in ranking is enormous. The authorship-weighted scheme is even more accurate because it is capable to distribute the authorship credit non-uniformly, for example, to allocate some extra credits to the first and/or corresponding authors. However, the analysis of real data shows that the improvement of the authorship-weighted scheme compared to the fractional scheme is not as high as one would expect. Hence, the first priority for fair ranking of researchers is to substitute the standard scheme by the fractional scheme in scientific publication databases as the Web of Science (Thomson Reuters) or the Scopus (Elsevier). At later stages, some simple authorship-weighted scheme, as described in this paper, can be adopted for more accurate evaluations.
